# Characterization of crop residues from false banana */Ensete ventricosum/* in Ethiopia in view of a full-resource valorization

**DOI:** 10.1371/journal.pone.0199422

**Published:** 2018-07-05

**Authors:** Hanna Berhanu, Zebene Kiflie, Isabel Miranda, Ana Lourenço, Joana Ferreira, Sisay Feleke, Abubeker Yimam, Helena Pereira

**Affiliations:** 1 School of Chemical and Bioengineering, Addis Ababa Institute of Technology, Addis Ababa, Ethiopia; 2 Centro de Estudos Florestais, Instituto Superior de Agronomia, Universidade de Lisboa, Lisbon, Portugal; 3 Ethiopian Environment and Forest Research Institute, Addis Ababa, Ethiopia; Monash University, AUSTRALIA

## Abstract

False banana /*Ensete ventricosum* [Welw.] Cheesman/ is exploited as a food crop in Ethiopia where it represents an important staple food. The plant is harvested and large amounts of biomass residues are originated, mainly from the pseudo stem (i.e., fiber bundles obtained from the leaf sheaths after being scrapped to produce starchy food) and the inflorescence stalk. These materials were studied in relation to their summative chemical composition, composition of lignin, lipophilic and polar extracts. Moreover, their structural characteristics, in view of their valorization, were scrutinized. The analytical studies were performed with the aid of FTIR, GC/MS, Py-GC/MS and SEM. The fiber bundles are aggregates of mainly long and slender fibers with low ash, extractives and lignin contents (3.8%. 4.4% and 10.5% respectively) and high holocellulose and α-cellulose contents (87.5% and 59.6% respectively). The hemicelluloses in the fibers are mostly highly acetylated xylans and the lignin is of the H-type (H:G:S, 1:0.7:0.8). This lignin composition is in line with the FTIR peaks at 1670 cm^-1^ and 1250 cm^-1^.The inflorescence stalk has high ash content (12.3% in the main stalk and 24.6% in fines) with a major proportion of potassium, high extractives (25.9%), and low lignin and α-cellulose contents (5.8% and 17.9% respectively). The stalk includes numerous starch granules in the cellular structure with the predominant presence of parenchyma. The potential valorization routes for these materials are clearly different. The fiber bundles could be used as a fiber source for paper pulp production with the possibility of a prior hemicelluloses removal while the inflorescence stalk has nutritional value for food and fodder. Furthermore, it can also be used for sugar fermentation products.

## 1. Introduction

**Fig 1:**
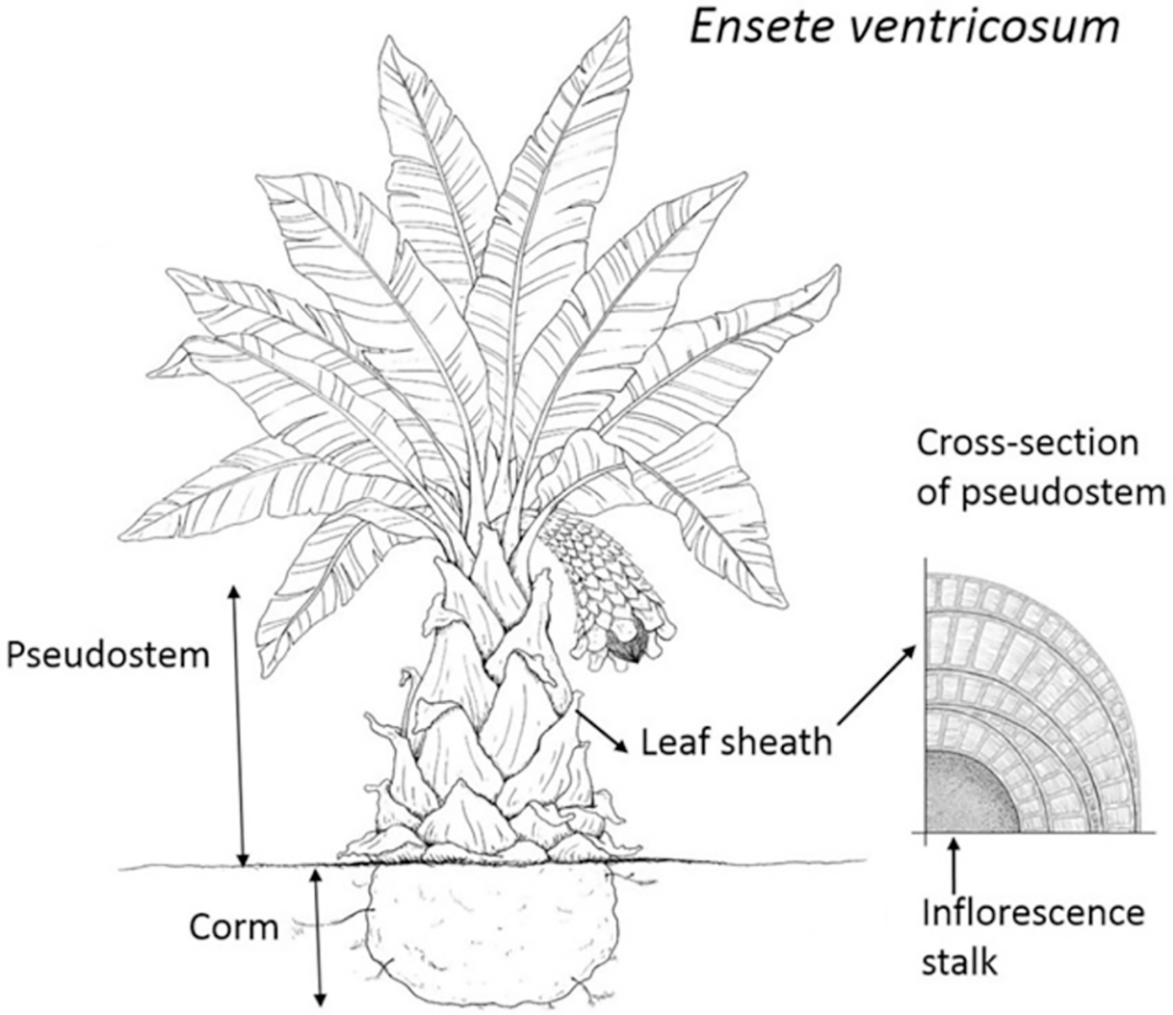
***Ensete* ventricosum plant (left) and cross-section of enset pseudo stem (right)**.

Enset /*Ensete ventricosum* [Welw.] Cheesman/ is a monocarpic tall perennial herbaceous plant which belongs to the order Scitamineae, family Musaceae and genus Ensete. It is commonly named as false banana because it is very similar and a close relative to the banana plant (*Musa* sp.) although the enset fruit is not edible. The plant has an adventitious root system, an underground stem structure known as corm, a pseudo stem that is dilated at the base and large leaves. The above ground part of enset pseudo stem is formed by a bundle of clasping and overlapping leaf sheaths (Fig 1). As the plant grows older and matures, an inflorescence grows at the apex and a stalk develops along the inner part of the pseudo stem, and the flower emerges at the top and droops out.

The height of an enset plant may range from 4 to 13 m. The pseudo stem has a circumference ranging from 1.5 to 3.0 m and a length of 2 to 5 m. The leaves are 4 to 6 m long and 0.6 to 0.9 m wide [1]. The corms are 0.7–1.8 m long and 1.5–2.5 m in diameter at maturity [2]. The proportion of the different components of the enset plant (% dry matter) was reported as lamina and midribs of leaves 15–17%, leaf sheaths 45–51%, stalk 9–11% and corm 26–29% [3] but a higher variation range was reported among different varieties e.g. leaf lamina 6–16%, leaf midribs 4–21%, pseudo stem 46–60% and corm 10–30% [4].

Enset is a drought resistant plant that is cultivated in over 200,000 hectares in the highlands (1100–3000 m a.s.l) of central, southern and south-western parts of Ethiopia. Average temperature of 10–21°C is conducive for the growth of the plant [5, 6]. In addition, wild enset is common in central, eastern and southern Africa and Asia [4, 7].

The enset plantations are usually harvested after the appearance of the inflorescence, at an age of about 6–7 years. However, the plant can be harvested at any stage if there is shortage of food or cattle feed. Enset provides a staple food for over 20% of the Ethiopian population living in the southern and south-western regions from its pseudo stem and corm, while it is hardly known as a food plant elsewhere [6, 8, 9]. It is estimated that enset-based foods provide double to 20 times more calories than cereals [10, 11].

The corm as well as a starchy pulp obtained by scraping the lower part of the leaf sheath are used in different popular nourishing specialties that have nutritional value and antioxidant properties, namely *kocho*, *bulla*, *amicho* [12]. *Kocho* is a fermented product from the scrapped parenchymatic tissue of leaf sheaths and pulverized corm. *Bulla* is a dehydrated mixture of the juice from the scrapped leaf sheaths, pulverized corm and grated stalk of the inflorescence. *Amicho* is the boiled stripped corm of the younger plants [13].

**Fig 2:**
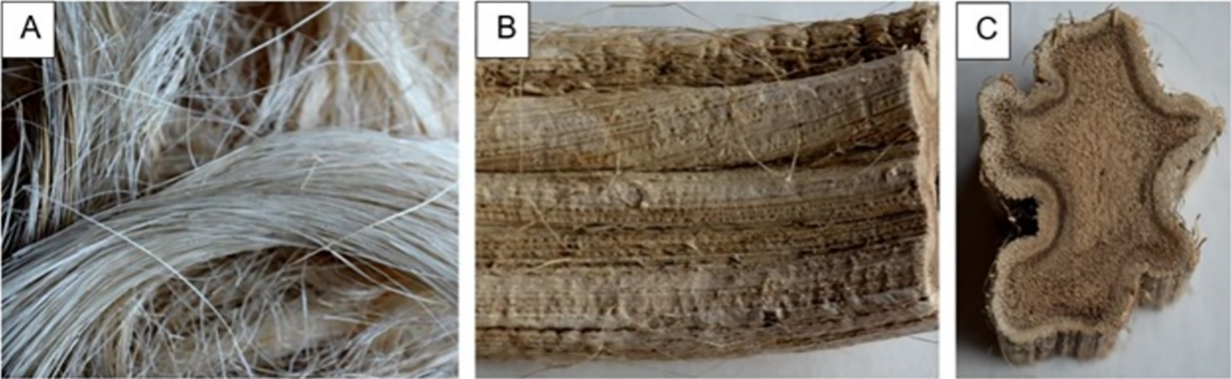
**Enset fiber bundle (A), longitudinal view of stalk (B) and cross sectional view of stalk (C)**.

Enset food crop produces several residual by-products, mainly fibers from the pseudo stem and the inner inflorescence stalk (Fig 2). In fact, the scraping of the parenchymateous tissue of the lower sheath part of the leaves that make up the pseudo stem, gives a solid residue of a fibrous nature, commonly named fibers, that is used traditionally to make sacks, bags, ropes, mats, and sieves [6, 11]. These fibers are cellular clusters with diameters ranging from 100 to 400 μm and have been studied regarding their tensile behaviour pertaining to their industrial application as natural fibers and as reinforcement in composite materials [14, 15]. However, currently large quantities of these residues are available without significant commercial value.

In this work, the enset crop residual products remaining from the food production are analyzed in view of their valorization. Although some reports exist on the mineral and nutritional value of enset plant parts mainly targeting their use as livestock feed [3, 16, 17], detailed chemical composition with regard to their valuation is not known. This study was focused on both the fibers obtained from the scraping of the pseudo stem as well as the inflorescence stalk remaining after leaves sheath removal were collected. The structural features of the materials were observed and their chemical composition thoroughly analyzed in terms of lignin, polysaccharides, extractives and ash, including a detailed analysis of extractives and lignin composition. The overall objective was to design valorization procedures for these residual materials that will strengthen the local economies and provide full-resource crop utilization.

## 2. Materials and methods

### 2.1. Sampling

Two types of residues, obtained from the exploitation of enset /*Ensete ventricosum* [Welw.] Cheesman/ for food production were used in this study: the fibers obtained from the scraping of the leaf sheaths and the inflorescence stalk (Fig 2). The samples were collected from private enset plantation located in Wolkite, Gurage zone, Ethiopia (08^0^17’N; 37^0^47’E, 1920 m of altitude) upon verbal permission of the owners. However, specific permission was not required from the ethical committee. The study did not involve endangered or protected species.

The enset fibers and stalks were fractionated separately using a knife mill (Retsch SM 2000) with an output sieve size of 6 mm^2^ and 10 mm^2^, respectively. The particle size distribution was determined by using a vibratory sieving apparatus (Retsch AS 200 basic) for 10 min shaking time with different sieve sizes.

Graphical description of the methodology is included in supplementary material (S1 Fig).

### 2.2. Summative chemical composition

The chemical summative analysis was carried out using 40–60 mesh (0.250 to 0.450 mm) particle size fractions. All the chemical determinations were made on duplicate samples.

The inflorescence stalk was analyzed separately in two fractions: the main fraction (here called stalk main) and the fines (called stalk fines). For chemical analysis, the main fraction of the stalk was further pulverized to obtain particles between 40–60 mesh.

Ash content was calculated gravimetrically according to TAPPI standard method (T211 om-93). Extractives were determined by successive extraction of samples using dichloromethane, ethanol and water for 6 h, 16 h and 16 h, respectively, based on TAPPI T 204 om-88. The extractives solubilized by each solvent were determined gravimetrically and reported as percent of the original sample.

The Klason lignin and acid soluble lignin were determined based on TAPPI T222 om-98 and TAPPI UM 250, respectively. 350 mg of extractive free sample was treated with 72% sulfuric acid at 30 ^o^C for 1 h, where after it was diluted to 3% and autoclaved for 2 h at 120 ^O^C and 1.2 bar. The solid residues were filtered, oven dried and weighed as Klason lignin. The filtrates were used for acid soluble lignin determination by UV-spectrophotometer at the wavelength of 250 nm. The monosaccharaides including neutral sugars and uronic acids as well as acetates were quantitatively determined in the hydrolysis liquor by High Performance Anion Exchange Chromatography.

The holocellulose content of extractive-free samples was determined by the chlorite method [18]. One gram of sample was placed in an Erlenmeyer flask and 32 ml of distilled water was added. While slowly shaking, 0.750 g of NaClO_2_ and 0.3 ml of acetic acid were added and the flask was covered with watch glass and boiled at 70°C for 1 h. This procedure was repeated three times. After cooling, the sample was filtered using filter flask and washed with 50% cold water and acetone until sample was free of acid. The insoluble portion was dried at 105°C for 4 h, cooled in a desiccator and weighed repeatedly until a constant weight was obtained.

The α-cellulose was determined by treating 250 mg of the holocellulose with 1.25 ml of 17.5% NaOH and stirring with glass rod. In 5 min intervals, 0.6 ml of 17.5% NaOH was added two times. After 30 min the fiber suspension was diluted with 6.6 ml of distilled water and was filtered with a coarse crucible. Finally, it was washed thoroughly with 12.5 ml of 8.3% NaOH and 100 ml distilled water, and soaked in 1.9 ml of 10% acetic acid for 5 min. The neutralized α-cellulose was washed with distilled water and dried at 105°C and the yield calculated in relation to the original material. The hemicelluloses fraction was calculated by difference between holocellulose and α-cellulose content.

### 2.3. Composition of lipophilic extractives

The lipophilic extracts that were solubilized with dichloromethane were recovered after solvent evaporation and dried under vacuum at room temperature overnight. Aliquots (2 mg) were taken and derivatized prior to analysis. The samples were dissolved in 100 μL of pyridine and the compounds with hydroxyl and carboxyl groups were trimethylsilylated into trimethylsilyl (TMS) ethers and esters, respectively, by adding 100 μL of bis(trimethylsily)-trifluoroacetamide (BSTFA). The reaction mixture was then heated at 60°C for 30 min and immediately analyzed by GC–MS by injecting in a GC–MS Agilent 5973 MSD with the following conditions: Zebron 7HG-G015-02 column (30 m, 0.25 mm ID, 0.1 μm film thickness), injector 280°C, oven temperature program 50°C (1 min), rate of 10°C min^-1^ up to 150°C, rate of 4°C min^-1^ up to 300°C, rate of 5°C min^-1^ up to 370°C, rate of 8°C min^-1^ up to 380°C (5 min). The MS source was kept at 220°C and the electron impact mass spectra (EIMS) was taken at 70 eV of energy.

The compounds were identified as TMS derivatives by comparing their mass spectra with a GC–MS spectral NIST and WIELY library and by comparing their fragmentation profiles with published data. For semi-quantitative analysis and a full scan to find all possible compounds, the area of peaks in the total ion chromatograms of the GC–MS analysis was integrated and their relative proportions expressed as area percentage. Each aliquot was injected in triplicate and the results are presented as their mean.

### 2.4. Phenolic content and anti-oxidant properties of polar extracts

Approximately 0.5 g of samples was extracted with 20 ml of 1:1 ethanol-water mixture for 60 min at 50°C in an ultrasonic bath. The insoluble material was oven dried at 60°C overnight and at 105°C for 2 h, and the extraction yield was calculated as percent mass loss of the initial material. The filtrates were stored at 4°C for further analysis of phenolic extract composition and antioxidant activity.

The total amount of soluble phenolic was estimated by the Folin–Ciocalteu method using gallic acid as standard [19]. Total flavonoids were quantified by an aluminium chloride colorimetric assay using catechin as a standard [20] and the proanthocyanidins content (condensed tannins) determined by the vanillin-H_2_SO_4_ method using catechin as a standard [21]. The experimental procedure is described in detail in Sartori et al. [22].

The antioxidant activity was assessed by the DPPH free radical assay [23] following a procedure described before [22]. The antioxidant activity was expressed as IC_50_ values and also in terms of Trolox equivalents (TEAC).

### 2.5. Ash composition and nitrogen content

The ash obtained by combustion in a muffle furnace at 500°C was analyzed for macro- and micro-element concentrations. The ash was dissolved in HCl and the concentrations of Ca, Mg, Fe, Cu, Mn, Zn, Na, and K were determined by atomic absorption spectrophotometry in a Pye Unicam SP-9 apparatus (Cambridge, UK) equipped with a GF95 graphite furnace.

Nitrogen was determined by the Kjeldahl method [24] in detector equipment (Herdon, VA, USA) and was expressed as protein content using a conversion factor of 4.4.

### 2.6. FTIR spectroscopy

1 mg of extractive-free samples were ground with a Retsch MM200 mixer ball mill for 10 min and dried at 105°C for 24 h. Samples were analyzed in ATR mode FTIR in the 400 to 4000 cm^-1^ range with a resolution of 4 cm^-1^ and 64 scans accumulation.

### 2.7. Analytical pyrolysis (Py-GC/MS)

The extractive-free samples were dried and ground in a Retsch MM200 mixer ball mill for 10 min, and 0.20 mg of each sample was pyrolysed in a 5150 CDS apparatus fitted to an Agilent GC 7890B with a mass detector system 5977B installed with a ZB-1701 fused-silica capillary column (60 m x 0.25 mm i.d. x 0.25 μm film thickness). The chromatograph oven program was: 40 ^o^C held for 4 min, 10 ^o^C min^-1^ to 70°C, 5 ^o^C min^-1^ to 100 ^o^C, 3 ^o^C min^-1^ to 265 ^o^C, held for 3 min, 5 ^o^C min^-1^ to 270 ^o^C, held for 9 min. The compounds were identified by comparing their mass spectral fragmentation with Wiley and NIST2014 libraries. The peak molar area of each identified compound was calculated, summed and the percentage of each compound was calculated. The percentage of hydroxyl phenol (H), guaiacyl (G) and syringyl (S) derived products were separately summed and the S/G and H/G/S ratios were calculated.

### 2.8. Anatomical characterization

The samples were impregnated with DP1500 polyethylene glycol, and transverse sections of approximately 17 μm thicknesses were cut with a Leica SM 2400 microtome stained and mounted [25].

For fibers length determination, samples were macerated in 1:1 solution of 30% H_2_O_2_ and CH_3_COOH at 60°C for 48 h and stained with astra blue. A light microscope (Leica DM LA) was used, photomicrographs were taken with a digital camera (Leica DFC 320) and image acquisition was performed with Leica software Qwin V3.5.0 (Leica Micro systems Imaging Solutions, Cambridge, UK).

The samples were also observed by scanning electron microscope using a Hitachi-TM3030 Plus table top microscope.

## 3. Results

### 3.1. Structural characterization

**Fig 3:**
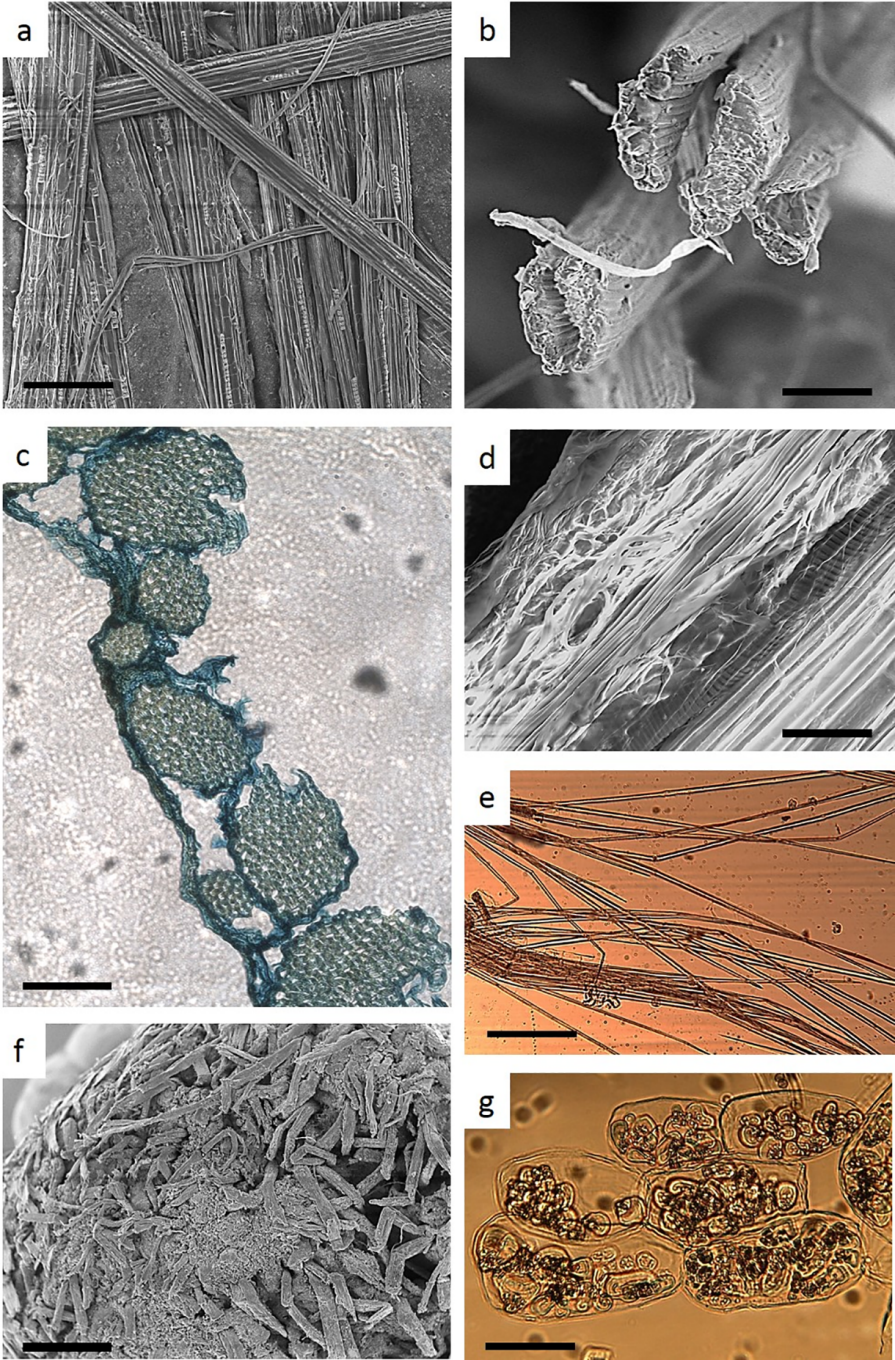
Structural features of enset fiber bundles and pseudo stem. (A) SEM longitudinal observation of various fiber bundles, scale bar 200 μm (B) SEM observation of the cross section of fiber bundles, scale bar 100 μm (C) optical microscopy observation of a thin cross section of one fiber bundle, scale bar 100 μm (D) SEM observation of a longitudinal cut of one fiber bundle; scale bar 50 μm (E) optical microscopy observation of dissociated fibers from one fiber bundle, scale bar 200 μm (F) SEM observation of a cross-section of the pseudo stem; scale bar 400 μm (G) optical microscopy observation of dissociated cells of the pseudo stem, showing starch granules scale bar 50 μm.

The fiber bundles show a yellowish white appearance with a rough surface and some stiffness (Fig 2A). Their cross-sectional view is roughly elongated to circular with approximately 0.1–0.2 mm diameter. SEM photographs show that each thread is a compact cellular cluster of mostly long cells (Fig 3A) that are contained by an enveloping membrane (Fig 3B). This was also seen in microscopic thin cross-sections and Fig 3C shows the disrupted membrane and fragments of the cellular aggregates. A longitudinal cut along the fiber thread shows different cell types, including the predominant fibers as well as vessels and parenchyma (Fig 3D).

Fiber length was measured from the cellular fibers obtained after dissociation of the bundles (Fig 3E). Only the fibers with the two ends clearly visible were considered for measurement. Results show that fibers length varied from 2.65 mm to 11.37 mm (mean 5.29 mm), width from 15.3 to 18.1 μm, lumen width from 7.0 to 11.2 μm, and cell wall thickness from 2.3 to 4.6 μm.

The stalk included an outer cortex layer and a central cylinder (Fig 2C) containing fibro-vascular bundles embedded in parenchyma (Fig 3F). The presence of starch in the parenchyma cells was very high featuring elongated to circular granules with dimension ranging approximately from 10 to 30 μm (Fig 3G).

### 3.2. Chemical composition

The chemical compositions of the enset fiber bundles obtained from scraping of the leaf sheaths, and of the inflorescence stalk are given in Table 1. The chemical compositions of both materials were very different.

**Table 1:** Summative chemical composition (mass % o.d. material) and monosaccharide composition (molar % of total monosaccharaides) of the enset fibers and stalks (stalk main and stalk fines).

	Fiber	Stalk main	Stalk fines
**Ash**	3.80	12.30	24.60
**Extractives**	4.44	25.92	25.01
**Dichloromethane**	0.43	1.05	0.81
**Ethanol**	1.11	7.43	5.95
**Water**	2.90	17.44	18.25
**Lignin**	10.53	5.75	3.39
**Klason lignin**	8.22	3.62	2.11
**Acid soluble**	2.31	2.13	1.28
**Holocellulose**	87.47	46.73	46.73
**α-cellulose**	59.59	17.03	17.03
**Protein**	0.66	3.78	3.21
**Monosaccharide (% molar)**			
**Arabinose**	1.29	5.37	3.46
**Galactose**	0.48	1.71	1.07
**Glucose**	51.87	80.59	89.94
**Xylose**	14.25	8.64	4.59
**Rhamnose**	0.00	0.00	0.00
**Mannose**	0.32	0.54	0.33
**Galacturonic acid**	1.29	0.91	0.63
**Glucuronic acid**	0.69	1.73	1.07
**Acetyl**	29.82	0.00	0.00

The fibers have a comparatively low content of extractives (4.4%), of which more than 90% were polar compounds that are soluble in ethanol and water. The lignin content was found to be around 10.5% and holocellulose and α-cellulose contents were high (87.5% and 59.6%, respectively). The monomeric composition of the polysaccharides (Table 1) consists mainly of glucose (51.9% molar proportion) while the hemicellulosic units comprise mainly xylose (14.3% molar proportion), a large proportion of acetyl groups (29.8% molar proportion) with smaller contents of arabinose, galactose, mannose and uronic acids.

The main inflorescence stalk has high extractives content (25.9%), mainly corresponding to polar compounds that were soluble in ethanol and water (Table 1). The lignin content was low (5.8%), as well as that of holocellulose (46.7%) and α-cellulose (17.0%). The monomeric composition of stalk polysaccharides is dominated by glucose (80.5% molar proportion), while the hemicellulosic units comprise xylose, arabinose, galactose, mannose and uronic acids, but no acetyl groups. The fines obtained from the inflorescence stalk had a similar composition to the main fraction with only a higher content of ashes (24.6%, Table 1).

### 3.3. Ash content and mineral composition

The ash content of the stalk was 12.3% in the main fraction and 24.6% in the fines while in the fibers it was only 3.8% (Table 1). The ash composition is summarized in Table 2. It is dominated by the presence of potassium in both materials but especially in the inflorescence stalk, while calcium, magnesium and sodium were present in similar and low amounts. In relation to micronutrients, the content of iron was very high in both materials [139–177 mg/kg].

**Table 2:** Elemental composition of the mineral component of the enset fibers and inflorescence stalk (stalk main and stalk fines).

Elements	Fiber	Stalk main	Stalk fines
**N, %**	0.15	0.86	0.73
**Na, %**	0.07	0.08	0.09
**K, %**	0.57	6.09	5.77
**Ca, %**	0.28	0.14	0.13
**Mg, %**	0.06	0.15	0.14
**Fe, mg/kg**	158.31	139.81	176.84
**Cu,mg/kg**	1.79	7.18	8.62
**Zn,mg/kg**	8.79	6.94	8.44
**Mn,mg/kg**	13.93	18.79	18.51

### 3.4. Lignin monomeric composition

**Fig 4:**
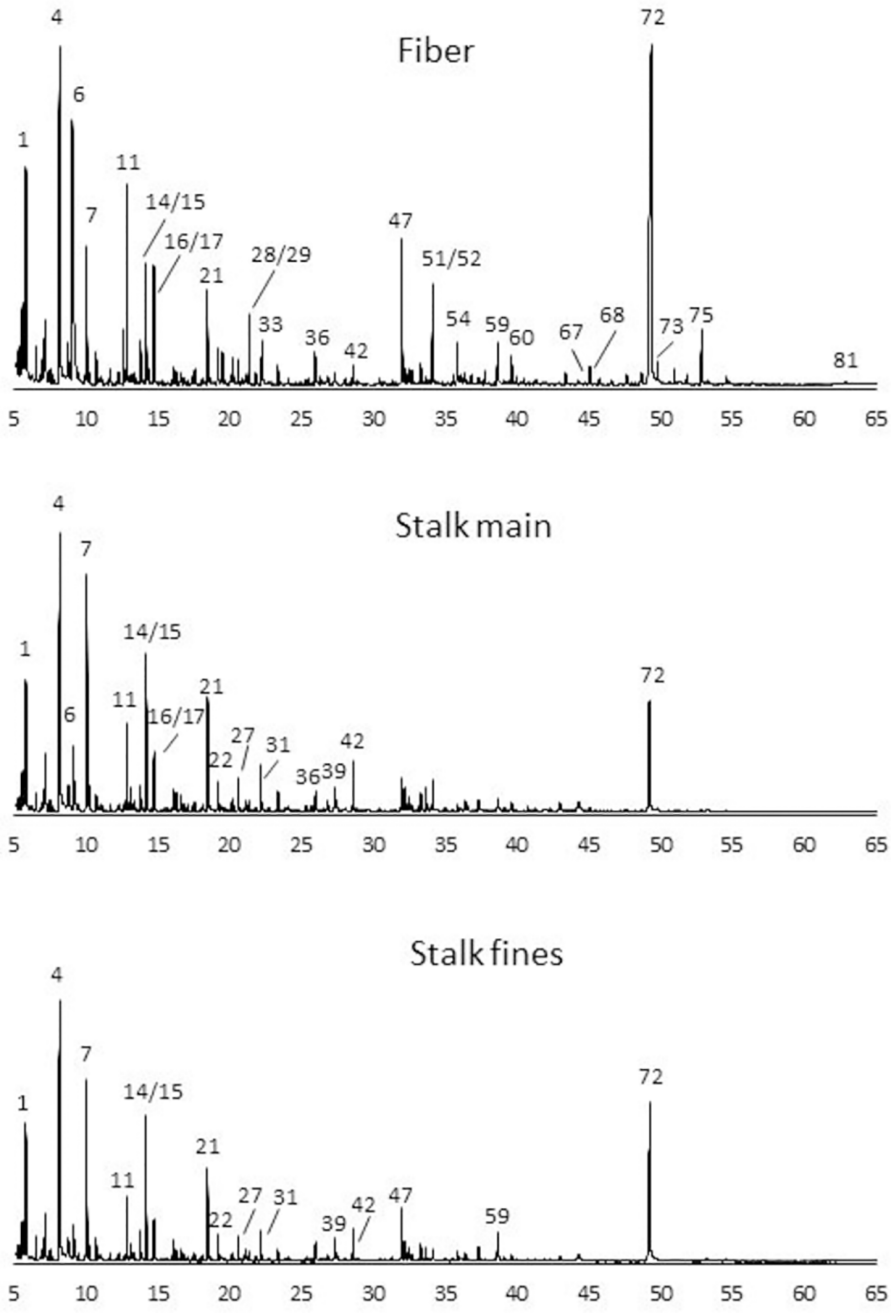
Pyrograms of the *Enset ventricosum* fiber bundles and inflorescence stalk (main fraction and fines). 1–2-oxo-propanal; 4 –hydroxyacetaldehyde; 6 –acetic acid; 7–1-hydroxy-propan-2-one; 11–3-hydroxypropanal; 14 –CH_3_-CO-CHOH-CHO; 15 –CHO-CH_2_-CH_2_-CHO; 16 –furfural; 17–2-cyclopenten-1-one; 21–2-hydroxy-2-cyclopenten-1-one; 22 –dihydro-methyl furanone isomer; 27 – 5*H*-furan-2-one; 28–4-hydroxy-5,6-dihydro-(2*H*)-pyran-2-one; 29–3-methyl-furan-2,5-dione; 31–2-hydroxy-3-methyl-2-cyclopenten-1-one; 33–2-hydroxy-1-methyl-1-cyclopentene-3-one; 36 –sugar derivative; 39–5-hydroxymethyldihydrofuran-2-one; 42 –sugar derivative; 47 –sugar derivative; 51–2,3-dihydrobenzofuran; 52–4-vinylguaiacol; 54 –similar to 4-allylphenol; 59–2-hydroxymethyl-5-hydroxy-2,3-dihydro-(4*H*)-pyran-4-one; 60 –methylindole; 67–4-vinylsyringol; 68–4-allylsyringol; 72–1,6-anhydro-β-D-glucopyranose; 73 –*trans*-4-propenylsyringol; 75 –Not identified; 81 –*trans*-sinapaldehyde.

The pyrograms of the enset fibers and inflorescence stalks are shown in Fig 4 and the list of the peaks with their quantitative proportion (% of total peak area) as well as the H:G:S and S: G ratios are given in Table 3.

**Table 3:** Peak identification, retention time, origin (C-carbohydrate, S-syringyl, G-guaiacyl, H-hydroxyphenyl, NPS-undetermined phenolic, P-protein) and quantification (% of total area of the chromatogram) of the pyrolysis products from *Ensete ventricosum* fiber bundle and inflorescence stalk (stalk main and stalk fines).

Peak	RT	Compound	Origin	Fiber	Stalk main	Stalk fines
**1**	5.82	2-oxo-propanal	C	4.6	4.6	5.6
**2**	7.11	Methyl vinyl ketone	C	0.67	0.50	0.61
**3**	7.18	Butane-2,3-dione	C	1.4	1.9	1.8
**4**	8.14	Hydroxyacetaldehyde	C	7.3	11.0	11.9
**5**	8.73	But-(E)-2-enal	C	0.70	0.86	0.90
**6**	9.08	Acetic acid	C	8.4	3.3	2.2
**7**	10.06	1-hydroxy-propan-2-one (acetol)	C	2.0	6.2	5.3
**8**	10.25	Toluene	NPS	0.23	0.79	0.58
**9**	10.72	Ethendiol	C	0.53	0.49	0.70
**10**	12.64	Ethane-1,2-diol	C	0.89	-	0.18
**11**	12.86	3-hydroxypropanal	C	2.5	1.9	1.6
**12**	13.82	2-oxo-3-en-butanal	C	0.44	0.45	0.56
**13**	13.82	3-furaldehyde	C	0.44	0.45	0.56
**14#**	14.19	3-oxo-2-ol-butanal	C	1.0	2.4	2.3
**15#**	14.19	Butandial	C	1.0	2.4	2.3
**16#**	14.76	Furfural	C	1.0	0.88	0.76
**17#**	14.76	2-cyclopenten-1-one	C	1.0	0.88	0.76
**18**	16.01	Furfuryl alcohol	C	0.31	0.82	0.79
**19**	17.49	4-cyclopentene-1,3-dione	C	0.21	0.28	0.24
**20**	17.64	Similar to 4-cyclopentene-1,3-dione	C	0.29	0.31	0.27
**21**	18.46	2-hydroxy-2-cyclopenten-1-one	C	1.4	3.1	2.8
**22**	19.2	Dihydro-methyl furanone isomer	C	0.67	0.82	1.05
**23#**	19.45	Unidentified sugar-derived	C	0.12	0.12	0.11
**24#**	19.45	5-methyl-2-furaldehyde	C	0.12	0.12	0.11
**25**	19.53	Sugar derived (m/z 55, 86, 114)	C	0.50	0.15	0.15
**26**	20.24	Dihydro-2(3*H*)-furanone (butyrolactone)	C	0.54	0.45	0.37
**27**	20.62	(5*H*)-furan-2-one	C	0.54	1.1	1.0
**28**	21.39	4-hydroxy-5,6-dihydro-(2*H*)-pyran-2-one	C	1.2	0.25	0.55
**29**	21.37	3-methyl-furan-2,5-dione	C	-	0.25	-
**30**	21.84	(2*H*)-pyran-2-one	C	0.26	0.19	0.22
**31**	22.15	2-hydroxy-3-methyl-2-cyclopenten-1-one	C	-	1.3	1.0
**32**	22.21	Methyl-dihydro-(2*H*)-pyran-2-one	C	0.46	-	-
**33**	22.28	2-hydroxy-1-methyl-1-cyclopentene-3-one	C	1.1	0.22	0.20
**34**	23.36	Phenol	H	0.36	0.67	0.42
**35**	24.16	Guaiacol	G	0.15	0.24	0.10
**36#**	25.96	Not identified sugar (overlapped spectra)	C	1.0	1.1	1.2
**37**	26.79	4-methylphenol (p-cresol)	H	0.18	0.36	0.21
**38**	26.88	3-methylphenol (m-cresol)	H	0.09	0.14	0.16
**39**	27.35	5-hydroxymethyl dihydrofuran-2-one	C	0.36	1.2	1.3
**40**	28.07	Levoglucosenone	C	0.10	-	-
**41**	28.32	4-methylguaiacol	G	0.05	-	-
**42**	28.59	Unidentified sugar-derived	C	0.40	1.7	1.3
**43**	28.72	2,4-dimethylphenol (2,4-Xylenol)	H	0.09	0.21	0.12
**44**	28.91	3,5-dihydroxy-2-methyl-(4*H*)-pyran-4-one	C	0.09	-	-
**45**	30.46	3-ethylphenol	H	0.17	0.18	0.09
**46**	30.74	Benzoic acid	NPS	0.09	-	-
**47**	32.01	Sugar derived (m/z 43, 57, 69, 82, 85, 96, 116)	C	2.9	-	2.0
**48**	32.20	Similar to dihydro-6-methyl-2H-pyran-3(4*H*)-one	C	0.35	0.81	0.78
**49**	33.33	1,4:3,6-dianhydro-α-D-glucopyranose	C	0.43	0.62	0.62
**50**	33.66	Sugar-derived (m/z 43, 57, 60, 68, 73, 86)	C	0.18	0.99	0.65
**51#**	34.15	2,3-dihydrobenzofuran (coumaran)	H	1.0	0.61	0.34
**52#**	34.15	4-vinylguaiacol	G	1.0	0.61	0.34
**53#**	35.84	5-hydroxymethylfurfural	C	0.85	0.22	0.61
**54#**	35.84	Similar to 4-allylphenol	H	0.21	-	-
**55#**	36.38	Syringol	S	0.24	0.11	0.09
**56#**	36.38	Unidentified sugar-derived	C	0.24	0.46	0.35
**57**	36.63	Indole	P	-	0.22	0.11
**58**	37.32	Dihydro-4-hydroxy-2-(3*H*)-furanone	C	-	0.47	0.61
**59**	38.68	2-hydroxymethyl-5-hydroxy-2,3-dihydro-(4*H*)-pyran-4-one	C	1.1	0.66	1.5
**60**	39.64	Methylindole	P	-	0.09	0.07
**61**	39.66	1,5-anhydro-arabinofuranose	C	0.71	0.37	0.30
**62**	40.02	4-methylsyringol	S	0.12	0.04	0.07
**63**	40.47	Vanillin	G	0.17	0.10	0.06
**64**	40.95	1-(4-hydroxy-3-methoxyphenyl)propyne	G	0.07	-	-
**65**	41.25	4-hydroxybenzaldehyde	H	0.04	0.18	0.07
**66**	41.36	1-(4-hydroxy-3-methoxyphenyl)propyne	G	0.08	-	-
**67**	45.11	4-vinylsyringol	S	0.34	0.18	-
**68**	45.78	4-allylsyringol	S	0.15	0.03	-
**69**	46.54	P-coumaric alcohol	H	0.11	-	-
**70**	47.69	*Cis*-4-propenylsyringol	S	0.25	-	-
**71**	49.02	4-propinylsyringol	S	0.09	-	-
**72**	49.31	1,6-anhydro-β-D-glucopyranose	C	17.1	5.4	11.4
**73**	49.82	*Trans*-4-propenylsyringol	S	-	0.10	-
**74**	51.01	Syringaldehyde	S	0.31	0.03	-
**75**	52.86	Unidentified	-	1.2	0.1	-
**76**	53.34	Acetosyringone	S	0.09	-	-
**77**	54.65	*Trans*-coniferaldehyde	G	0.09	-	-
**78**	54.78	Syringylacetone	S	0.08	-	-
**79**	55.84	Propiosyringone	S	0.02	-	-
**80**	56.33	Syringyl vinyl ketone	S	0.01	-	-
**81**	62.89	*ztrans*-sinapaldehyde	S	0.11	-	-
**Total carbohydrates**				67.4	61.8	69.5
**Total lignin**				6.1	4.6	2.7
**H**				2.3	2.3	1.4
**G**				1.6	0.9	0.5
**S**				1.8	0.5	0.2
**S/G**				1.1	0.5	0.3
**H:G:S**				1:0.7:0.8	1:0.4:0.2	1:0.4:0.1

The peaks corresponding to carbohydrate-derived compounds were dominant in all the pyrograms in comparison with the lignin-derived compounds. Levoglucosan (peak 72) was the major carbohydrate-derived compound representing 17.1% of the pyrolysis products of fibers, 5.4% of main stalks and 11.4% of stalk fines. Other carbohydrate-derived compounds were also found: 2-hydroxy-2-cyclopenten-1-one (peak 21) represented 1.4%, 3.1% and 2.8% respectively, 2-hydroxymethyl-5-hydroxy-2,3-dihydro-(4*H)*-pyran-4-one (peak 59) represented respectively 1.1%, 0.7%, 1.5%; and 4-hydroxy-5,6-dihydro-(2H)-pyran-2-one (peak 28) was higher in fibers (1.2%) and lower in stalks (0.3% and 0.6%). The low molecular weight compounds—hydroxyacetaldehyde (peak 4), acetic acid (peak 6), 1-hydroxy-propan-2-one (peak 7), 2-oxo-propanal (peak 1), 3-hydroxypropanal (peak 11)—represented 24.8%, 27.1% and 26.6% of total area for fibers, stalk and fines, respectively.

The pyrograms show clearly a lower abundance of lignin-derived peaks, which is in accordance with the lignin content determined by wet chemical analysis (Table 1). The peaks identified as lignin-derived were mostly from H-lignin units such as phenol (peak 34), p-cresol (peak 37), m-cresol (peak 38), 2,4-dimethyl-phenol (peak 43), 3-ethylphenol (peak 45), 2,3-dihydrobenzofuran (peak 51), a compound similar to 4-allyphenol (peak 54), 4-hydroxybenzaldehyde (peak 65) and *p*-coumaric alcohol (peak 69). The main compound derived from G-units was 4-vinylguaiacol (peak 52) with 1.0%, 0.6% and 0.3% of total area for fiber, stalk and stalk fines respectively. Several peaks were derived from S-units in lower proportion, they summed 1.8% and less than 0.5% in fibers and stalks respectively.

The monomeric composition of enset lignin, therefore, showed a predominance of H units: the H:G:S ratio was 1:0.7:0.8 for the fibers, 1:0.4:0.2 for the main stalk and a similar value of 1:0.4:0.1 for the stalk fines (Table 3). The S/G ratio was higher in the fibers (1.1), than in stalks (0.5 and 0.3 respectively).

The compounds indole (peak 57) and methylindole (peak 60) were also identified in the pyrograms of the stalks where they represented a small proportion of the total peak area (0.31% and 0.18% in stalks and fines respectively).

### 3.5. Lipophilic and ethanol-water extracts

The compositional profile of the lipophilic extracts from the fiber bundles and inflorescence stalk is given in Table 4 and shows little differences between the materials. The major fractions correspond to fatty acids, mainly saturated alkanoic acids (32–40% of the total compounds) that included mainly hexadecanoic acid, and substituted alkanoic acids (16–29%) including mainly 9-octadecenoic acid in the stalks and 9,12-octadecadienoic acid in the fibers. In addition, significant amount of glycerol derivatives were also present (16% in fiber and 25% in stalk), mainly β-sitosterol was the most important followed by stigmasterol and campesterol.

**Table 4:** Composition (% of total peak area) of TMS-derivatized dichloromethane extracts from *Ensete ventricosum* fibers and inflorescence stalk (stalk main and stalk fines).

Compounds	Fiber	Stalk main	Stalk fines
**Phenolics**	**1.97**	**-**	**-**
4-Hydroxybenzyl alcohol	0.30	-	-
4-Hydroxy-3-methoxybenzaldehyde	0.47	-	-
3,5-Dimethoxy-4-hydroxybenzaldehyde	0.41	-	-
4-Hydroxy-3-methoxybenzoic acid	0.42	-	-
3,5-Dimethoxy-4-hydroxybenzoic acid	0.37	-	-
**Alkanols**	**3.85**	**0.86**	**0.76**
1-Dodecanol	0.20	-	-
1-Tetradecanol	-	0.11	-
1-Hexadecanol	1.35	0.29	0.37
1-Heptadecanol	-	0.18	-
1-Octadecanol	1.22	0.28	0.39
1-Eicosanol	0.41	-	-
1-Tetracosnaol	0.67	-	-
**Saturated alkanoic acids**	**31.71**	**34.64**	**39.53**
Decanoic acid	0.24	0.10	-
Dodecanoic acid	0.34	-	-
Tetradecanoic acid	1.56	0.58	0.51
Pentadecanoic acid	1.97	1.18	0.87
Hexadecanoic acid	10.12	17.67	24.11
Heptadecanoic acid	1.67	1.09	0.87
Octadecanoic acid	3.98	6.23	10.98
Nonadecanoic acid	0.46	0.21	-
Eicosanoic acid	2.42	1.78	1.30
Heneicosanoic acid	1.88	1.01	-
Docosanoic acid	2.30	1.63	0.89
Tricosanoic acid	1.45	1.08	-
Tetracosanoic acid	2.22	1.81	-
Pentacosanoic acid	0.55	0.27	-
Hexacosanoic acid	0.55	-	-
**ω-Hydroxy alkanoic acid**	**-**	**0.2**	**-**
ω-Hydroxyoctadecanoic acid, methyl ester	-	0.2	-
**Substituted alkanoic acids**	**15.79**	**25.62**	**28.96**
*cis*-Eicosenoic acid	-	0.10	-
9-Tetradecenoic acid	0.70	-	-
13-Methyl-9-tetradecenoic acid	-	0.07	-
9-Hexadecenoic acid	2.40	1.61	1.15
10-Heptadecenoic acid	0.87	0.68	0.56
9-Octadecenoic acid	-	21.54	21.87
10-Nonadecenoic acid	-	0.10	-
9,12-Octadecadienoic acid	11.73	1.03	5.38
13-Docosenoic acid	-	0.49	-
4-Pentenoic acid	0.09	-	-
**Saturated alkanoic di acids**	**0.41**	**0.08**	**-**
Nonanedioic acid	0.41	0.08	-
**Glycerol derivatives**	**15.68**	**25.05**	**24.94**
Campesterol	2.09	3.51	3.98
Stigmasterol	3.73	5.68	4.79
β -Sitosterol	9.05	12.79	15.54
Fucosterol	0.70	0.82	-
Cycloartenol	-	0.80	-
ni sterol1	0.11	0.75	0.63
ni sterol2	-	0.19	-
ni sterol3	-	0.51	-
**Triterpenes/Triterpenoids**	**0.90**	**0.06**	**-**
Isochiapin B	-	0.06	-
Dehydroabietic acid	0.90	-	-
**Sugars**	**0.15**	**0.58**	**0.61**
ni sugar 1	0.15	-	-
ni sugar 2	-	0.15	-
ni sugar 3	-	0.10	-
ni sugar 4	-	0.33	0.61
**Other**	**2.71**	**0.35**	**-**
Sitosteryl-3β -D-Glucopyranoside	2.22	0.35	-
Campesteryl.3β -D-glucopyranoside	0.24	-	-
Stigmasteryl-3β -D-glucopyranoside	0.25	-	-
**TOTAL ID**	**82.49**	**94.8**	**98.59**
**TOTAL NI**	**17.51**	**5.2**	**1.41**
**TOTAL**	**100**	**100**	**100**

The characterization of the ethanol-water extracts (Table 5) shows that the content in phenolic compounds is very low and that they have no relevant antioxidant properties.

**Table 5:** Ethanol-water extraction yield, total phenolics, tannins, flavonoids and monosaccharides contents, and antioxidant activity of *Ensete ventricosum* fiber and inflorescence stalk (stalk main and stalk fibers).

	Fibers	Stalk main	Stalk fines
Extraction yield (%)	1.85	17.64	13.73
Total phenolics (mg GAE/g of extract)	38.22	17.61	23.01
Tannins (mg catechin/g of extract)	0	5.88	5.79
Hydrolysable tannins (mg of tannic acid/g of extract)	0	24.85	0
Flavonoids (mg catechin /g of extract)	19.2	5.34	44.42
Antioxidant capacity TEAC (mg Trolox/ g of extract)	142.11	22.255	23.41
IC_50_ value (μg extract /ml)	67.51	214.62	154.55
Total monosaccharides (mg/g of extract)	63.76	30.63	5.79

### 3.6. FTIR-ATR spectra

**Fig 5:**
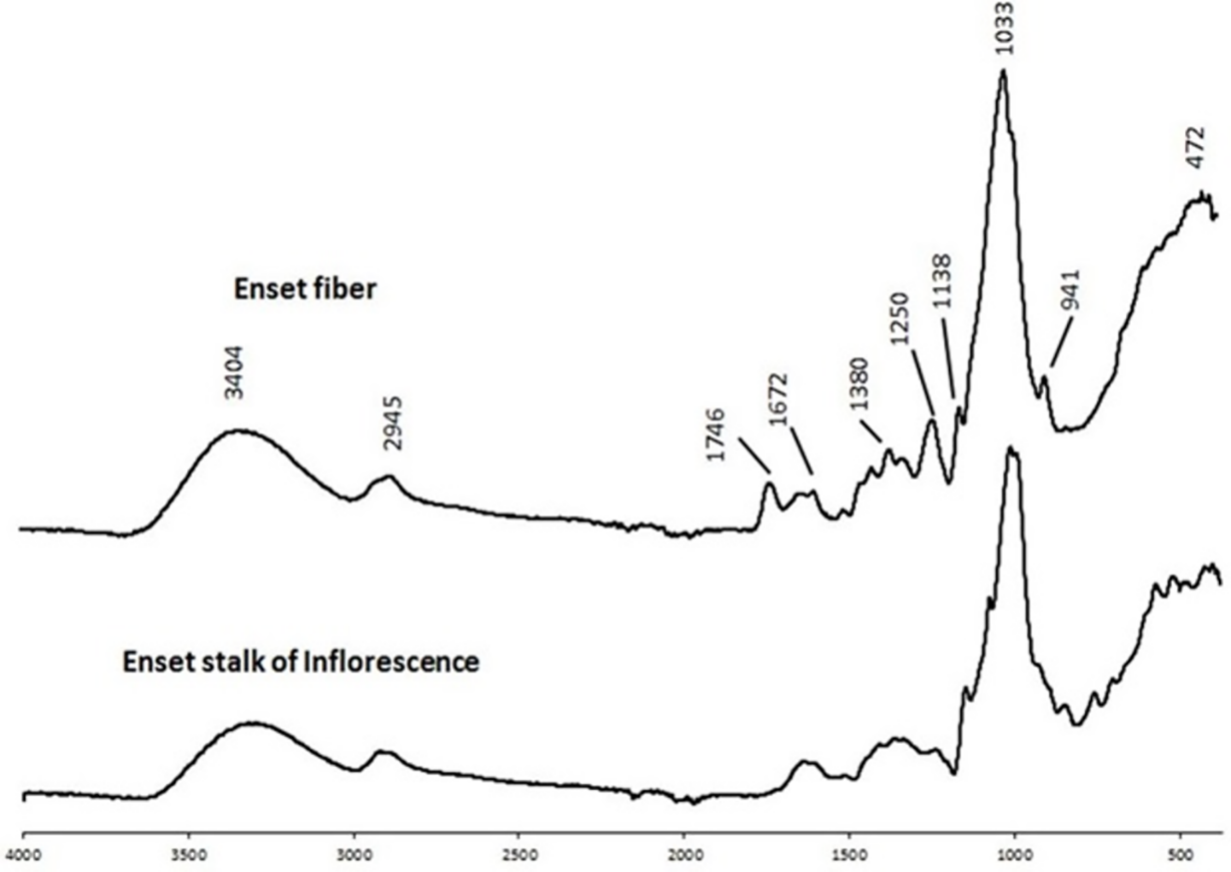
FTIR/ATR spectra of enset fiber bundles and enset inflorescence stalk.

The FTIR-ATR spectra for the extractive-free fiber bundles and inflorescence stalk are given in Fig 5. Some of the bands appeared in both materials: around 3400 cm^-1^, 2900 cm^-1^, 1740 cm^-1^, 1380 cm^-1^ and 1030 cm^-1^. The bands around 1672 cm^-1^ and 1250 cm^-1^ were only found in the spectra from the fibers.

## 4. Discussion

### 4.1. Structural characterization

The studied materials have very distinct characteristics (Fig 2) that show different application potential. The fiber bundles are compact cellular structures (Fig 3A–3D) corresponding to the fibro-vascular bundles of the leaf sheaths, as described in reference plant anatomy books [26]. The parallel lying fibers in the bundles (Fig 3A and 3D) indicate their ability for use as a fibrous source. One option could be the direct use of the bundles for rug and mat weaving, as it is already done traditionally, or as a fiber component in composite panel formulations as recently researched [14, 15, 27]. The fiber bundles that were mechanically characterized in those works showed similar cross-sectional dimensions (0.2 mm) and cellular structures as observed in the present work. Another option for the use of the fiber bundles may be their dissociation into individual fiber cells to be used for paper production, as it has been researched for the banana fibers [28, 29, 30].

The inflorescence stalk has a hard cortex around the central cylinder (Fig 2C) with a predominant presence of parenchyma where fibro-vascular bundles are embedded (Fig 3F). This structure is similar to that of floral stalks of other plants e.g. of *Cynara cardunculus* [25], but a remarkable feature is the presence of high amounts of starch granules (Fig 3G). This fact together with other chemical features allow considering a nutrition related application, as further discussed.

### 4.2. Ash content and mineral composition

The inflorescence stalk had high mineral content and the fines obtained upon grinding were enriched in minerals showing double ash content, as shown in Table 1. It is known that ash tends to accumulate in the finest sized fraction during biomass processing due to the small size and brittleness of the inorganic materials [31, 32].

**Fig 6:**
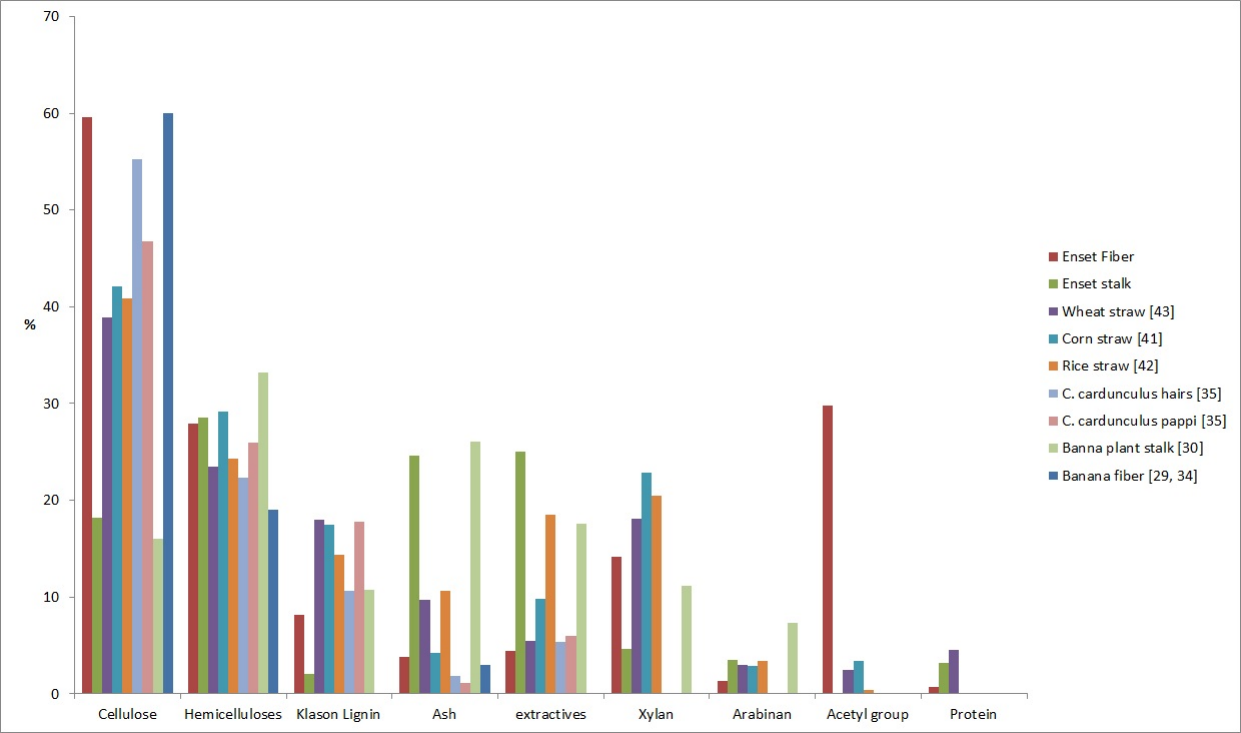
Graphical comparison of enset fiber bundle and inflorescence stalk chemical composition with other biomass sources.

There are a few reports on the ash content in different parts of enset, which are similar to the results obtained here, mainly related to its use as food and fodder: leaf sheaths 6.4%, stalk 15.3% [3], pseudo stem 8.8% and 7.5% [4, 33]. The reported higher ash content value in the leaf sheath than in the fibers may be explained by removal of leaf sheath components during the scraping procedure (Table 1). High ash content was also found for stalk and leaf sheath of banana plant [30] while lower ash content was reported for fiber bundles isolated from the banana pseudo stem, similar to what was found in the present work [34]. C. cardunculus hairs and pappis had even lower ash content than enset fiber bundles, as shown in Fig 6 [35].

The mineral components of stalk and fibers consisted mainly of potassium while among the micronutrients iron was present in considerable amount (Table 2). This has been reported in enset pseudo stem as well as in other plant parts (K 2.1–5.2%) [16]. The mineral composition is similar to that given for floral stalks and leaf sheaths of the banana plant with values for the different elements in the same order of magnitude [30]. It is interesting to notice that the mineral composition of enset and banana biomass components is very specific in the high K and Fe contents. In contrast, the floral stalk of *Cynara cardunculus* has only 0.9% K and 0.06% Fe [36].

The mineral compositional profile and the high ash content of the inflorescence stalks makes them interesting mineral nutrient providers with potential application in food and feed.

### 4.3. Chemical composition

The chemical composition of the enset fiber bundles and that of the inflorescence stalk are very different (Table 1) in proportion and composition of the structural and non-structural components. The corresponding results for these two materials will be discussed separately.

The fibers are characterized by very high holocellulose content and the large proportion is α-cellulose. The hemicelluloses are mainly xylans; considering the molar composition given in Table 1, for 100 hemicellulosic sugar units (without glucose), xylose corresponds to 81 units, uronic acids to 13 units, arabinose to 7 units and mannose and galactose to 5 units. The hemicelluloses have a high acetylation: 1.7 acetyl groups per sugar monomer were detected.

A comparison with the fibers extracted from the banana pseudo stems shows somewhat comparable hemicellulose, holocellulose and α-cellulose compositions as represented in Fig 6 [36, 37].

The enset fibers have moderate lignification (Table 1) and the lignin showed a predominance of H units: the H:G:S ratio was 1:0.7:0.8 (Table 3). This is the first time that enset lignin composition is reported. The proportion of H-units in enset lignin is overwhelming in the macromolecular structure and above values reported on lignin in other grasses. The presence of H-units in lignin is characteristic of herbaceous plants although the composition varies with species: the H:G:S ratio of corn, wheat and rice straws being 1:8.8:15.3, and 1:9.8:9.2, 1:3.0:2.6, respectively [38]. Higher proportion of H-units is found in *Musa* species: in abaca fibers [*Musa textilis*] the lignin has a H:G:S ratio of 1:0.7:2.1 [39] while in the banana plant [*Musa acuminata* Colla var. *cavendish*] the H:G:S ratio of 1:1.0:0.2 in leaf sheaths nears the values obtained here for the enset lignin [30].

With regard to the inflorescence stalk, the chemical composition of the main fraction and of the fines was similar, except for the ash content as already discussed. The stalk had high extractives content and low lignin, holocellulose and α-cellulose content (Table 1). The sugar units obtained by hydrolysis were predominantly glucose that comprised over 80% of the molecules. This means that a considerable proportion of glucose should result from the hydrolysis of starch which is conspicuously present in the enset stalk (Fig 3G). Enset is a starch producing plant and enset starch has high proportion of the branched amilopectin component [40]. The hemicelluloses in the enset inflorescence stalk were mostly xylans but in comparison with the fibers had higher proportion of arabinose and no acetyl groups were found (Table 1). It had lower xylan content than that of corn straw [41], rice straw [42] and wheat straw [43] with comparable arabinan content (Fig 6).

Overall chemical composition of the inflorescence stalk of enset is to some extent similar to that of stalk of banana plant with lower lignin content as shown in Fig 6 [29, 30]. For banana leaf sheaths, Li et al. [37] reported 72.7% holocellulose and 39.1% cellulose and carbohydrate composition of glucose 71.8%, xylose 11.2%, arabinose 7.3%, galactose 2.0%, mannose 0.6%, galacturonic acid 7.1%. The predominance of xylose units in the composition of hemicelluloses in banana leaf sheaths was also reported [29, 30]. Starch is present also in the banana plant floral stalks in amounts of 26.3% [30].

The enset stalk lignin is mostly H-lignin (Table 3) with a predominance of H units much above that of the enset fibers. The only available comparison can be made with the banana plant floral stalk and that shows a H:G:S ratio of 1: 1.9:0.4 [30].

The pyrogram of extractive-free enset fibers and stalk were dominated by carbohydrate-derived peaks. Levoglucosan was the compound representing the major proportion of the pyrolysis products of fibers, stalks and stalk fines, as shown in Table 3. This is the common feature in the pyrolysis of carbohydrates and lignocellulosic materials [44]. Other carbohydrate-derived compounds were 2-hydroxy-2-cyclopenten-1-one; 2-hydroxymethyl-5-hydroxy-2,3-dihydro-(4*H*)-pyran-4-one; and 4-hydroxy-5,6-dihydro-(2*H*)-pyran-2-one. The presence of the pyran structure in the pyrolysis products reflects the higher content of xylans in the fibers (Table 1) since this is an indicator for xylans [45]. The compounds indole and methylindole are protein-derived peaks [45] and their presence in stalks and absence in fibers is in accordance with the protein content in these plant parts.

The enset stalk contains 3% protein, as shown by the determination of organic nitrogen (Table 1) as well as by pyrolysis data (Table 4). Crude protein content of various parts of enset plant has been reported from 3.7% to 13.2% [33] which is therefore considered of nutritional value and used for human food and for fodder. Similar amounts of protein (range of 2–8%) were also reported for the banana plant [32, 46].

The FTIR spectral features of the extractive free enset fiber and stalk (Fig 5) show similar peaks around 3400 cm^-1^, 2945 cm^-1^, 1746 cm^-1^, 1380 cm^-1^ and 1033 cm^-1^ which correspond to different functional groups of cellulose, hemicelluloses and lignin [47, 48]. These bands were also found in different parts of the banana plant [30, 49]. The peaks around 1670 cm^-1^ and 1250 cm^-1^ are originated from S and G units of lignin respectively, and were noticeable in the fibers. This result supports the data obtained from lignin pyrolysis since the predominance of H unit is more pronounced in the stalk.

It is clear from the different structural (Figs 2 and 3) and chemical features (Tables 1–4) that the two residual materials from the exploitation of the enset crop have different potential applications and should have different valorization routes.

The fact that fiber bundles have higher cellulose content and long cellular elements, i.e. the fibers, makes them a candidate for pulping. However, although present in moderate amounts, the lignin has a monomeric composition with low syringyl content that will not be removed under mild reactive conditions and therefore the need for strong delignification conditions is to be expected. Advantages of enset fibers in relation to other herbaceous plants are the low ash and extractives content (Table 1). In complement, the high hemicelluloses content may require the use of pre-treatments for their removal, for instance by water thermal treatments (auto-hydrolysis) as proposed for various straws and other crop residues [41, 42]. Auto hydrolysis of enset fiber can be used under a bio refinery frame work for production of oligosaccharides in general, and of xylose oligosaccharides in particular due to their high xylan content [42, 43].

On the contrary, the inflorescence stalk has a high mineral content that may have nutritional value (Table 2), protein, a large proportion of extractives, a high content in glucan polymers, including starch, and little lignin (Table 1). Therefore, its use for food and feed can be an option, and valorization routes may include fermentation processes e.g. for ethanol. Moreover, the inflorescence stalk can produce enzymes such as amylolytic and lignocelluloytic enzymes by using microorganisms, as already tried for banana waste [50]. The enset inflorescence stalk may therefore integrate a valorization flow sheet as proposed by Panda et al. [51] for producing bio commodities from agricultural wastes.

### 4.4. Extractives content and composition

The enset inflorescence stalk contained substantially more extractives than the fiber bundles that were mostly solubilized by polar solvents (Table 1). Floral stalk of banana plant also showed high extractives content similar to enset inflorescence stalk, as shown in Fig 6 [30]. Herbaceous plants, like rice straw and *C*. *cardunculus* often have a large content of extractives (Fig 6) [42, 52].

The ethanol-water extracts yielded low content of phenols compounds (Table 5) and the antioxidant capacity was low; this indicates that these extracts are mainly composed of carbohydrates, as observed in water-soluble extracts from different morphologic regions of the banana plant [30].

The lipophilic extracts included mainly fatty acids and their derivatives; sterols were present but triterpenes appeared in very low amounts. This is the first time that the composition of enset lipophilic extracts is reported. The composition is very similar to that obtained for the banana plant which showed a predominance of saturated and unsaturated acids, mostly of hexanoic acid and 9,12- octadecadienoic acid, accompanied by a large content of sterols and little presence of aromatics [52]. Steryl glucosides that were present in the enset extracts from the fibers bundles (Table 4) were also found in the banana plant [52].

Although lipophilic extractives in fibrous materials may cause problems if pulp production is envisaged, in the enset fiber bundles they were present in very low contents (0.4%), therefore without significance for the formation of stickies in the pulp (Table 1).

In the case of the enset floral stalk, the lipophilic compounds are present in higher amounts (1.1%) as shown in Table 1 and they will add to the nutritional value of the material.

## 5. Conclusions

The biomass residues remaining from the exploitation of the food crop *Ensete ventricosum* as fiber bundles and inflorescence stalk have different structural and chemical features that point out to diverse valorization routes.

The fiber bundles are aggregates mainly of long and slender fibers with low ash, extractives and lignin contents, and high holocellulose and α-cellulose contents. These features show the possibility of delignification to produce paper pulps but taking into account that the high content of H-units in the lignin structure may need strong pulping conditions. The hemicelluloses, mostly highly acetylated xylans, may be removed prior to delignification for xylan-derived added-value products.

The inflorescence stalk has a nutritional value for food and fodder, since it contains high amounts of potassium and iron, protein and non-cellulosic carbohydrates associated to low lignin content. It may be used also for sugar fermentation products such as ethanol.

The enset pseudo stem residual components remaining after plant harvest and processing for food that were here chemically characterized for the first time, show potential for valorization thereby allowing a more complete resource use and an improvement of the crop economy.

## Supporting information

S1 Fig Flow chart describing the methodology used in the study. (TIFF)

## References

[1] Smeds H. The enset planting culture of eastern Sidamo, Ethiopia. Acta Geogr. 1955; 13: 1–39.

[2] Tsegaye A, Westphal E. *Ensete ventricosum* (Welw.) Cheesman. Record from PROTA4U Oyen L.P.A.; Lemmens R.H.M.J. [Editors]. PROTA (Plant Resources of Tropical Africa / Resources végétales de l’Afrique tropicale), 1992 Wageningen, Netherlands

[3] Fekadu D, Ledin I. Weight and chemical composition of the plant parts of enset /*Ensete ventricosum/* and the intake and degradability of enset by cattle. Livestock Production Science. 1997; 49: 249–257.

[4] Nurfeta A, Eik LO, Tolera A, Sundstøl F. Chemical composition and *in sacco* dry matter degradability of different morphological fractions of 10 enset /*Ensete ventricosum/* varieties. Animal Feed Science and Technology. 2008; 146: 55–73.

[5] Baker RED, Simonds NW. The genus of *Ensete* in Africa. KEW Bull. 1953; 8: 405–416.

[6] Brandt S, Spring A, Hiebsch C, McCabe J, Tabogie T, Diro M. The tree against hunger: Enset based agricultural systems in Ethiopia, Washington: American Association for the Advancement of Science 1997.

[7] Shumbulo A, Gecho Y, Tora M. Diversity, challenges and potentials of enset /*Ensete ventricosum*/ production: In case of a woreda, Wolaita Zone, Southern Ethiopia. Food science and Quality Management. 2012; 7: 24–31.

[8] Tsehaye Y, Kebebew F. Diversity and cultural use of enset */Enset ventricosum (*Welw.) Cheesman/ in Bonga in situ conservation site, Ethiopia. Ethnobotany Research and Applications. 2006; 4:147–157.

[9] Tsegaye A, Struik P. Enset /*Ensete Ventricosum* (Welw.) Cheesman/ kocho yield under different crop establishment methods as compared to yield of other carbohydrate-rich food crop. Netherlands journal of Agriculture Science. 2001; 49: 81–94

[10] Quinlan R, Quinlan M, Dira S, Caudell M, Sooge A, Assoma A. Vulnerability and resilience of Sidama enset and maize farms in Southwestern Ethiopia. Journal of Ethnobiology. 2015; 35(2): 314–336

[11] Gabel M, Karlsson LM. Nutritive values of the drought tolerant food and fodder crop *enset*. African Journal of Agricultural Research. 2013; 8(20): 2326–2333.

[12] Forsido S, Rupasinghe HPV, Astatkie T. Antioxidant capacity, total phenolic and nutritional content in selected Ethiopian staple food ingredients. International Journal of Food Sciences and Nutrition. 2013; 64(8): 915–920. doi: 10.3109/09637486.2013.806448 23777527 10.3109/09637486.2013.806448

[13] Ayalew A, Yeshitila M. The response of enset /*Ensete ventricosum* (Welw) Cheesman/ production to rate and frequency of N and P nutrients application at Areka, in Southern Ethiopia. Innovative Sys Des Engin. 2011; 2:26–32.

[14] Mizera C, Herák D, Müller M, Hrab P. Mechanical behavior of polymeric composite with fibers of false banana */Ensete ventricosum/*. Agronomy Research. 2015; 13: 680–689.

[15] Mizera C, Herák D, Hrabe P, Muller M, Kabutey A. Effect of length of false banana fibers /*Ensete ventricosum/* on mechanical behavior under tensile loading. Sciencia Agriculturae Bohemica. 2016; 47 (2): 90–96.

[16] Nurfeta A, Tolera A, Eik LO, Sundstøl F. Yield and mineral content of 10 enset */Ensete ventricosum/* varieties. Trop. Anim. Health Prod. 2008; 40: 299–309. 18557193 10.1007/s11250-007-9095-0

[17] Zewdie S, Mats Olsson M, Fetene M. Effect of drought/irrigation on proximate composition and carbohydrate content of two enset /*Ensete ventricosum* (Welw) Cheesman/ clones. Ethiop. J. Sci. 2008; 31(2):81–88.

[18] Rowell R.M. Handbook of Wood Chemistry and Wood Composites 2nd ed Boca Raton: Taylor and Francis; 2005.

[19] Singleton VL, Joseph AR. Colorimetry of total phenolics with phosphomolybdic-phosphotungstic acid reagents. Am J Enol Vitic. 1965; 16: 144–158

[20] Zhishen J, Tang M, Wu J. The determination of flavonoid contents in mulberry and their scavenging effects on superoxide radicals. Food Chemistry. 1999; 64: 555–559

[21] Abdalla S, Pizzi A, Ayed N, Bouthoury FC, Charrier B, Bahabri F, et al MALDI-TOF analysis of Aleppo pine */Pinus halepensis/* bark tannin. BioResources. 2014; 9: 3396–3406. doi: 10.15376/biores.9.2.3396–3406

[22] Sartori C, Mota GS, Ferreira J, Miranda I, Mori FA, Pereira H. Chemical characterization of bark of *Eucalyptus urophylla* hybrids in view of their valorization in bio refineries. Holzforchung. 2016; 70(9): doi: 10.1515/hf-2015-0258

[23] Sharma OP, Bhat TK. DPPH antioxidant assay revisited. Food chemistry. 2009; 113 (4): 1202–1205. doi: 10.1016/j.foodchem.2008.08.008

[24] Jackson ML. Soil Chemical Analysis. Englewood Cliffs: Prentice- Hall; 1958.

[25] Quilhó T, Gominho J, Pereira H. Anatomical characterization and variability of the thistle *Cynara cardunculus* in view of pulping potential. IAWA Journal. 2004; 25(2): 217–230.

[26] Fahn A. Plant Anatomy. 2nd ed Oxford: Pergamon Press; 1974.

[27] Mizera C, Herák D, Hrab P, Müller M, Kabutey A. Mechanical behavior of *Ensete ventricosum* fibers under tendion loading. Journal of Natural Fibers. 2017; 14: 187–196.

[28] Rahman MM, Islam T, Nayeem J, Jahan MS. Variation of chemical and morphological properties of different parts of banana plant /*Musa paradisiac/* and their effects on pulping. International Journal of Lignocellulosic Products. 2014; 1(2): 93–103.

[29] Cordeiro N, Belgacem MN, Torres IC, Moura J.C.V.P. Chemical composition and pulping of banana pseudo-stems. Industrial Crops and Products. 2004; 19: 147–154. doi: 10.1016/j.indcrop.2003.09.001

[30] Oliveira L, Cordeiro N, Evtuguin DV, Torres IC, Silvestre A.J.D. Chemical composition of different morphological parts from ‘Dwarf Cavendish’ banana plant and their potential as a non-wood renewable source of natural products. Industrial Crops and Products. 2007; 26: 163–172. doi: 10.1016/j.indcrop.2007.03.002

[31] Bridgeman TG, Darvell LI, Jones JM, Williams PT, Fahmi R, Bridgwater AV, et al Influence of particle size on the analytical and chemical properties of two energy crops. Fuel. 2007; 86:60–72. doi: 10.1016/j.fuel.2006.06.022

[32] Liu X, Bi X T. Removal of inorganic constituents from pine barks and switch grass. Fuel Process. Technol. 2011; 92: 1273–1279. doi: 10.1016/j.fuproc.2011.01.016

[33] Mohammed B, Gabel M, Karlsson L M. Nutritive values of the drought tolerant food and fodder crop enset. African J. Agric. Res. 2013; 8(20): 2326–2333.

[34] Bhatnagar R, Gupta G, Yadav S. A Review on Composition and Properties of Banana Fibers. International Journal of Scientific & Engineering Research. 2015; 6 (5)

[35] Gominho J, Lourenco A, Curt M, Fernandez J, Pereira H. Characterization of hairs and pappi from *Cynara Cardunculus capituala* and their suitability for paper production. Industrial Crops and products. 2009; 29(1): 116–125. doi: 10.1016/j.indcrop.2008.04.022

[36] Cordeiro N, Oliveira L, Faria H, Belgacem MN, Moura J.C.V.P. Surface modification of banana-based lignocellulose fibers. Contact Angle, Wettability and Adhesion. 2006; 4: 387–405.

[37] Li K, Fu S, Ahan H, Zhan Y, Lucia LA. Analysis of the chemical composition and morphological structure of banana pseudo-stem. Bio Resources. 2010; 5(2): 576–585.

[38] Buranov AU, Mazza G. Lignin in straw of herbaceous crops. Industrial Crops and Products. 2008; 28: 237–259.

[39] Del Río J C, Gutiérrez A, Rodríguez I M, Ibarra D, Martínez A T. Composition of non-woody plant lignins and cinnamic acids by Py- GC/MS, Py/TMAH and FT-IR. J. Anal. Appl. Pyrol. 2007; 79:39−46. http://dx.doi.org/10.1016/j.jaap.2006.09.003

[40] Wondimu A, Molla F, Dinda SC, Gebre S N, Tadese E. Literature review on enset starch: Physico-chemical properties and pharmaceutical applications. Journal of Drug Delivery & Therapeutics. 2014; 4:1–6.

[41] Moniz P, Pereira H, Quilhó T, Carvalheiro F. Characterization and hydrothermal processing of corn straw towards the selective fractionation of hemicelluloses. Industrial Crops and Products. 2014; 50: 145–153. URL: http://dx.doi.org/10.1016/j.indcrop.2013.06.037

[42] Moniz P, Pereira H, Duarte LC, Carvalheiro F. Hydrothermal production and gel filtration purification of xylo-oligosaccharides from rice straw. Industrial Crops and Products. 2014; 62: 460–465. doi: 10.1016/j.indcrop.2014.09.020

[43] Carvalheir F, Silva-Fernandes T, Duarte L, Girio F. Wheat straw Auto hydrolysis: Process optimization and product characterization. Applied Biochem Biotechnol. 2009; 153: 84–93 19082765 10.1007/s12010-008-8448-0

[44] Lourenço A, Gominho J, Marques AV, Pereira H. Py-GC/MS (FID) assessed polysaccharides behaviour during Kraft delignification of *Eucalyptus globulus* heartwood and sapwood. Journal of Analytical and Applied Pyrolysis. 2013; 101: 142–149.

[45] Ralph J, Hatfield RD. Pyrolysis-GC-MS characterization of forage materials. *J* Agric Food Chem. 1991; 39: 1426–1437. doi: 10.1021/jf00008a014

[46] Bilba K, Arsene M A, Ouensanga A. Study of banana and coconut fibers. Botanical composition, thermal degradation and textural observations. Bioresource Technology. 2007; 98: 58–68. doi: 10.1016/j.biortech.2005.11.030 16442281 10.1016/j.biortech.2005.11.030

[47] Ramadevi P, Sampathkumar D, Srinivasa C V, Bennehalli B. Effect of alkali treatment on water absorption of single cellulosic abaca fiber. BioResources. 2012; 7(3): 3515–3524.

[48] Chen H, Ferrari C, Angiuli M, Yao J, Raspi C, Bramanti E. Qualitative and quantitative analysis of wood samples by Fourier transform infrared spectroscopy and multivariate analysis. Carbohaydrate Polymers. 2010; 82: 772–778.

[49] Oliveira L, Evtuguin D V, Cordeiro N, Silvestre A J D. Structural characterization of stalk lignin from banana plant. Industrial Crops and Products. 2009; 29: 86–95.

[50] Panda SK, Mishra SS, Kayitesi E, Ray RC. Microbial-processing of fruit and vegetable wastes for production of vital enzymes and organic acids: Biotechnology and scopes. Environmental Research. 2016; 146: 161–172. doi: 10.1016/j.envres.2015.12.035 26761593 10.1016/j.envres.2015.12.035

[51] Panda SK, Ray RC, Mishra SS, Kayitesi E. Microbial-processing of fruit and vegetable wastes into potential bio-commodities: a review. Critical Reviews in Biotechnology. 2017; 38: 1–16. doi: 10.1080/07388551.2017.1311295 28462596 10.1080/07388551.2017.1311295

[52] Oliveira L, Freire C S R, Silvestre A J D, Cordeiro N, Torres I C, Evtuguin D V. Steryl glucosides from banana plant *Musa acuminate Colla Var*. *Cavendish*. Industrial Crops and Products. 2005; 22:187–192

