# Supplementary figures and images for: Characterization of crop residues from false banana /Ensete ventricosum/ in Ethiopia in view of a full-resource valorization
Source: PLoS One. 2018 Jul 5;13(7):e0199422. doi: 10.1371/journal.pone.0199422 (PMC6033405; doi:10.1371/journal.pone.0199422)

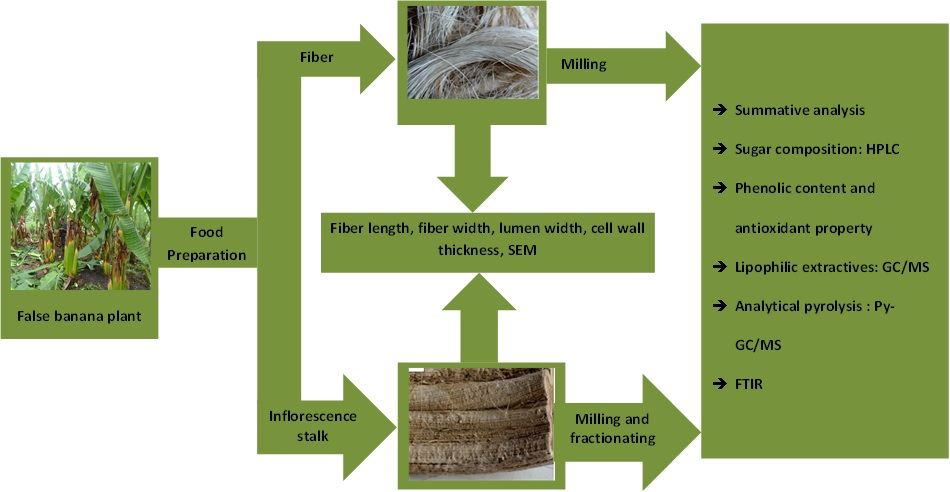

Supplement: S1 Fig — (TIFF) [file pone.0199422.s001.tiff]
